# From Polymeric Nanoformulations to Polyphenols—Strategies for Enhancing the Efficacy and Drug Delivery of Gentamicin

**DOI:** 10.3390/antibiotics13040305

**Published:** 2024-03-28

**Authors:** Ance Bārzdiņa, Aiva Plotniece, Arkadij Sobolev, Karlis Pajuste, Dace Bandere, Agnese Brangule

**Affiliations:** 1 Department of Pharmaceutical Chemistry, Riga Stradins University, 21 Konsula Str., LV-1007 Riga, Latvia; aiva@osi.lv (A.P.);; 2 Baltic Biomaterials Centre of Excellence, Headquarters at Riga Technical University, LV-1007 Riga, Latvia; 3 Latvian Institute of Organic Synthesis, 21 Aizkraukles Str., LV-1006 Riga, Latvia; arkady@osi.lv (A.S.); kpajuste@osi.lv (K.P.)

**Keywords:** gentamicin, antibacterial activity, drug delivery, nanoparticles, polyphenols, natural products, synergy

## Abstract

Gentamicin is an essential broad-spectrum aminoglycoside antibiotic that is used in over 40 clinical conditions and has shown activity against a wide range of nosocomial, biofilm-forming, multi-drug resistant bacteria. Nevertheless, the low cellular penetration and serious side effects of gentamicin, as well as the fear of the development of antibacterial resistance, has led to a search for ways to circumvent these obstacles. This review provides an overview of the chemical and pharmacological properties of gentamicin and offers six different strategies (the isolation of specific types of gentamicin, encapsulation in polymeric nanoparticles, hydrophobization of the gentamicin molecule, and combinations of gentamicin with other antibiotics, polyphenols, and natural products) that aim to enhance the drug delivery and antibacterial activity of gentamicin. In addition, factors influencing the synthesis of gentamicin-loaded polymeric (poly (lactic-co-glycolic acid) (PLGA) and chitosan) nanoparticles and the methods used in drug release studies are discussed. Potential research directions and future perspectives for gentamicin-loaded drug delivery systems are given.

## 1. Introduction

Due to the alarming rate of increase in antibacterial resistance, common broad-spectrum antibiotics often fail to treat infections. More and more often opportunistic and nosocomial pathogens, especially bacterial strains with a tendency to form biofilms, like *Pseudomonas aeruginosa*, are the cause of high-morbidity and high-mortality infections that require high-dosage and longer antibacterial treatments [1,2,3]. This again leads to increased antibacterial resistance, creating a vicious cycle. The majority of nosocomial infections are caused by six multidrug-resistant bacteria (*Enterococcus faecium*, *Staphylococcus aureus*, *Klebsiella pneumoniae*, *Acinetobacter baumannii*, *Pseudomonas aeruginosa,* and *Enterobacter* species). This group of bacteria is commonly referred to as ESKAPE pathogens [4]. All of these bacteria can also be found on the World Health Organization’s (WHO) priority list for research on and the development of new antibiotics for antibiotic-resistant bacteria at either critical or high-priority levels [5]. As there has been a serious shortage of new antimicrobials entering the market in recent years, a search for ways to enhance the efficacy and drug delivery of already known and widely used antibacterial agents like gentamicin is essential.

**Figure 1:**
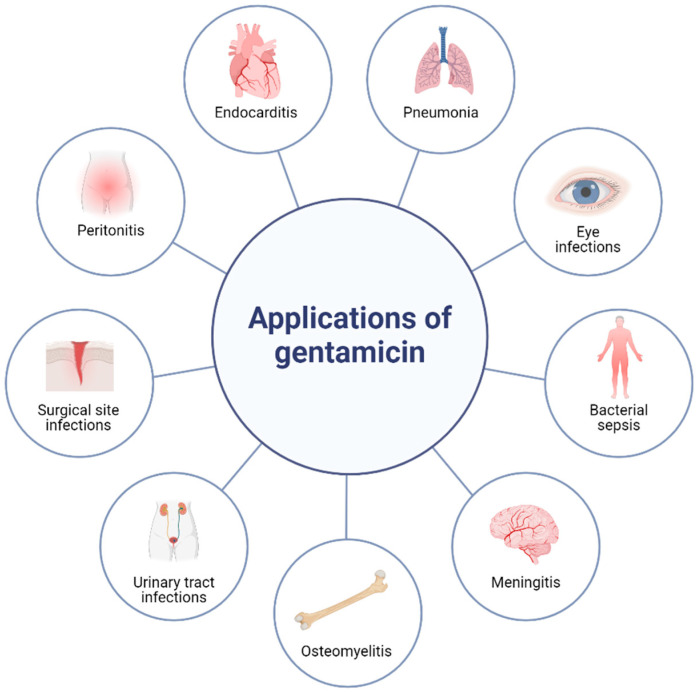
Applications of gentamicin. Created with BioRender.com (accessed on 29 February 2024).

Gentamicin is a broad-spectrum, bactericidal, aminoglycoside antibiotic. It is effective against a wide range of aerobic Gram-negative bacteria, as well as Gram-positive *Staphylococcus* species [6]. Of special importance is its potential activity against ESKAPE pathogens. Although it was discovered in the early 1960s [7], gentamicin still is a part of the essential medicines list of the World Health Organization [8]. Because of its wide activity spectrum, gentamicin is used to treat over 40 different clinical conditions, including bacterial sepsis, peritonitis, meningitis, the urinary tract and respiratory tract, eye, ear, bone, and surgical site infections, and others, proving its important role in modern medicine [6,8,9,10]. Figure 1 summarizes United States (US) Food and Drug Administration (FDA) approved, prevalent indications treated with gentamicin.

Gentamicin represents class III of the Biopharmaceutical Classification System due to its high solubility in water and low cellular penetration [11]. Consequently, drug delivery of this active agent alone may be hindered. As gentamicin is used to fight serious infections, often caused by multi-drug-resistant pathogens, ways to lower the needed concentration of gentamicin and ultimately minimize the chance of the development of resistance against it are one of the top priorities. 

Therefore, this review paper provides an overview of the chemical and pharmacological properties of gentamicin and offers six different strategies (the isolation of specific types of gentamicin, encapsulation in polymeric nanoformulations, hydrophobization of the gentamicin molecule, and combinations of gentamicin with other antibiotics, polyphenols, and natural products) that aim to enhance the drug delivery and antibacterial activity of gentamicin. 

## 2. The Components and Properties of Gentamicin Complex

**Figure 2:**
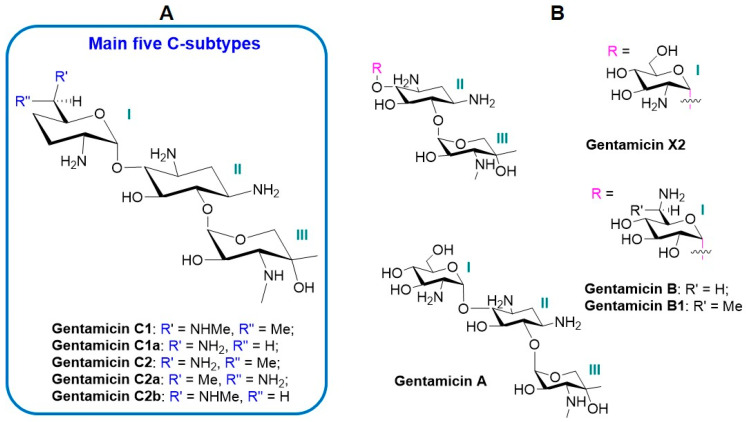
Structures of Gentamicin derivatives: (**A**): main C-subtypes; (**B**): minor components.

Gentamicin is produced by the *Micromonospora* species of bacteria, as a mixture of multiple components, but mainly consists of five structurally different C-subtypes (C1, C1a, C2, C2a, and C2b) with structural modifications at position 6′ of moiety I (Figure 2A) [12]. The structural differences in the major components are related by the level of methylation—a methyl or hydrogen substitution in two R groups on the 2-amino-hexose residue (I). In gentamicin C1a, methyl groups are missing, whereas both in C1 and C2, a methyl group is present at the 6′ position; gentamicin C1 is N-methylated at this position, while C1a and C2 have free amines. The C2 component consists of two stereoisomers [13]. The ratio of the gentamicin components varies depending on the drug’s manufacturing method, the fermentation conditions, and the purification procedure [14,15].

The major components constitute 92–99% of the gentamicin complex. Other gentamicin derivatives (Figure 2B), as the minor components, are found in the range between 0.8 and 5.3% [15,16]. 

According to the European Medicine Agency (EMA), the gentamicin composition consists of 10–30% C1a, 25–45% C1, and 35–55% C2, C2a, and C2b [17,18]. The ratio of gentamicin components has been studied using several methods based on paired-ion, high-performance liquid chromatography (HPLC) [19,20] and coupled liquid chromatography–NMR to compare the composition of different batches from various sources [15].

The clinical mechanism of action of the mixture is unclear; it is unknown whether the broad spectrum of antimicrobial action is due to multiple components. In other terms, it is unclear whether each component works across a narrower range of bacterial strains or species than the entire mixture [21]. Numerous research groups worldwide are working on the characterization of the structure–activity relationships of gentamicin components, studying the activity of the difficult-to-separate components of the commercial gentamicin samples, and characterizing specially prepared single components [21,22]. The relative toxicity and activity of the individual components in the commercial gentamicin samples have been studied since the 1970s [16,17]. These studies suggest that gentamicin C2 is less nephrotoxic than the other gentamicin constituents [23]; gentamicin C1 and C1a are less ototoxic than C2 [24]. In more recent studies evaluating the ototoxicity, it was indicated that gentamicin C1a was less ototoxic than a commercial gentamicin mixture. The minimum inhibitory concentration (MIC_50_ and MIC_90_) values of both the gentamicin complex and gentamicin C1a against clinical isolates of five bacterial species from *E. coli*, *K. pheumoniae*, *A. baumannii*, *P. aeruginosa*, and *S. aureus* were comparable; however, a two-fold increase in MIC_50_ was observed for the gentamicin C1a isomer compared to the gentamicin complex. However, these two compounds showed rather different in vitro activities against a larger portion of 61 isolates from *E. coli*, *K. pheumoniae*, *A. baumannii*, *P. aeruginosa*, and *S. aureus* [25].

Further research on the activity–toxicity relationship of the gentamicin components is necessary to draw concrete conclusions regarding the potential gains of isolating and applying individual gentamicin subtypes. 

## 3. Pharmacology, Stability, and Administration of Gentamicin

Gentamicin belongs to the 4,6-disubstituted-2-deoxystreptamine class of aminoglycosides [26]. Clinically, gentamicin has been used as a sulfate salt. It contains five basic nitrogens and requires five sulfuric acid equivalents per mole of every gentamicin base [27]. 

The mechanism of action prevents the synthesis of bacterial proteins through electrostatic binding with negatively charged phospholipids’ head groups. Afterwards, the antibiotic binds to the specific ribosomal proteins, resulting in the formation of inactive complexes that cause misreadings in mRNA [28,29]. Gentamicin is a cationic antibiotic with a narrow therapeutic index [30,31]. Its antibacterial activity can be influenced by the pH. A low pH is associated with a decrease in activity [30]. The pharmacological action of gentamicin is concentration-dependent [6]. 

The pharmacokinetics of gentamicin are comparable to those of other aminoglycosides. Gentamicin is a polar molecule and is poorly absorbed when taken orally (less than 1%); therefore, systemic use requires parenteral administration [31,32,33]. In certain infections, topical and local administration is needed [34]. The local delivery of antibiotics might reduce potential toxic side effects and be of particular use in infections that require the long-term, sustained release of antibiotics, like osteomyelitis [35]. 

It has been suggested that the standard intravenous gentamicin starting dose of 7 mg/kg based on total body weight appears to optimize the chance of reaching the exposure target after the first administration in both adults and children older than 1 month, including critically ill patients. However, despite numerous recent population pharmacokinetic studies, the optimal pharmacokinetic–pharmacodynamic target for efficacy is still unclear [36]. If patients suffer from renal failure, the dosage of gentamicin needs to be lowered [37]. 

Gentamicin binds to serum proteins very poorly; in most cases, the binding to serum proteins is under 15% [38]. In the cerebrospinal fluid of patients with uninflamed meninges, it is found in very low concentrations [37]. Gentamicin does not easily penetrate cells; instead, it is mostly distributed in the extracellular fluid [33]. Pinocytosis is the main mechanism of free gentamicin uptake [39]. Gentamicin does not undergo metabolism in the body, and, through glomerular filtration, unchanged gentamicin is quickly eliminated [33,40]. Since gentamicin has a short plasma half-life of around 2 h, frequent administration is needed [31]. The biodistribution of gentamicin causes its most common side effects—nephrotoxicity and ototoxicity [41,42]. The half-life in the renal cortex is estimated to be around 100 h [43]. The pharmacokinetics of gentamicin are dependent on the overall health status of the patient. Altered pharmacokinetic parameters like the volume of distribution, peak concentrations, and renal clearance are affected by conditions like sepsis, heart failure, peritonitis, and renal impairment [33,44].

Gentamicin sulfate sterile solutions should be kept between 2 and 8 °C. Gentamicin solutions have proven to be stable when kept at room temperature and in boiling aqueous buffers with pH values ranging from 2 to 14 [27,45]. Recommendations under the auspices of the International Society for Peritoneal Dialysis (ISPD) underlined that gentamicin is stable for 14 days at all temperatures [46]. A protocol used for microbe–epithelium cocultures states that gentamicin is stable at 4 °C for 1 month [47]. The good stability of gentamicin over a wide range of storage conditions makes it a promising candidate for encapsulation in novel drug delivery systems like nanoparticles. 

## 4. Polymeric Nanoformulations of Gentamicin

In the last two decades, nanoformulations have emerged as one of the frontrunners for the encapsulation and delivery of a wide range of active agents, including antibiotics. Nanoformulations for enhanced drug delivery have provided formulation scientists with the opportunity to achieve a higher bioavailability and biocompatibility, a controlled release, and increased target selectivity [48]. Although the use of nanoformulations in terms of their toxicity and distribution in the body is still under investigation, the potential gains of this approach drive further research in this field [14].

In the case of antibiotics, the main hope for the use of nanoformulations is that they will lower the chance of the development of antimicrobial resistance and achieve the targeted delivery of antibiotics with the lowest effective dose to avoid unwanted side effects for the patient [49,50]. For more than two decades, various lipids, polymers, and other materials have been applied to create micro- and nanoformulations, including nanoparticles, nanofibers, nanocomposites, and others, for the delivery of a wide range of antibiotics [50]. Gentamicin is no exception. In addition to polymeric nanoformulations, gentamicin has been successfully encapsulated into liposomes [51,52,53] and mesoporous silica nanoparticles [54,55,56], as well as being tested for synergic effects in combination with metallic nanoparticles [57,58,59]. A detailed overview of all nanoformulation types for the delivery of gentamicin has been thoroughly evaluated in a recent review paper by Athauda et al. [14]. The use of lipid-based nanoparticles for the delivery of antibiotics and the treatment of infections has already been reviewed in detail by Ferreira et al. [60,61]. Therefore, this review will only focus on recent advances in polymeric formulations, in particular, poly (lactic-co-glycolic acid) (PLGA) and chitosan-based systems, their potential applications, and the main variables impacting the systems and their kinetics. The sustained release properties of gentamicin-loaded nanoformulations and the possibility of enhancing the bioavailability of gentamicin are of particular importance; therefore, these topics will be discussed in detail in further subsections.

### 4.1. Gentamicin-Loaded PLGA Nanoparticles

PLGA is a polyester copolymer composed of lactic acid (PLA) and glycolic acid (PGA) in varying ratios. It is biodegradable, biocompatible, non-toxic, approved for parenteral use by both FDA and EMA, and allows for a wide range of surface modifications [62]. An overview of gentamicin-loaded PLGA nanoparticles and their main parameters can be found in Table 1.

Since gentamicin is highly hydrophilic, its penetration into cells might be hindered and the clearance of the drug is fast [11,31]. Hence, higher doses should be administered more frequently. In the case of gentamicin, the chance of developing side effects increases accordingly [31]. Low cellular penetration is especially associated with intracellular infections [64]. The encapsulation of gentamicin in polymeric nanoparticles like PLGA and chitosan could offer a solution to the problems caused by the high water-solubility of gentamicin. Drug-loaded nanoparticles have an improved bioavailability and permanence time at the site of infection, in addition to offering a sustained release and protection of premature degradation [11]. Polymers offer a wide variability of parameters, like molecular weight and the ratio between monomers, which can be applied to develop nanoformulations with optimal drug release properties.

In comparison to other polymers, like chitosan and alginate, PLGA nanoparticles can have controlled release properties without a chemical modification [67]. The sustained release of gentamicin from PLGA nanoparticles may be the most important advantage compared to using a free drug. The ratio between PLA and PGA is crucial to the hydrophobicity of the formulation [70]. A higher lactic acid content increases the hydrophobicity of the system and prolongs the release of drugs [64,70]. In addition, a sustained release of the drug can also be achieved by using a polymer with a higher molecular weight [71]. In case of chronic, long-lasting infections, a sustained release will determine the efficacy of the therapy. To date, PLGA 50:50 is the most commonly used PLA:PGA ratio in nanoformulations due to its favorable physiochemical properties and release kinetics [72].

**Figure 3:**
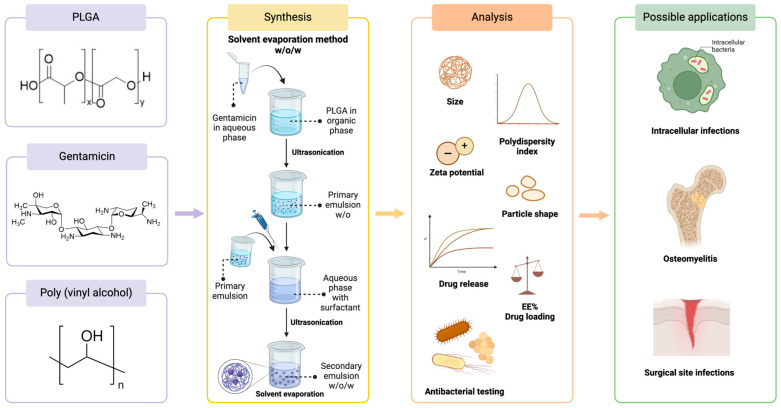
Synthesis, common characterization methods, and possible applications of gentamicin-loaded PLGA nanoparticles. Created with BioRender.com (accessed on 21 March 2024).

The small molecular weight and the high water-solubility of gentamicin make it challenging to encapsulate it into nanocarriers [11]. One key advantage of using PLGA as a nanocarrier is its ability to encapsulate both hydrophobic and hydrophilic substances. However, different formulation methods need to be applied. For a hydrophilic drug (like gentamicin) encapsulation, multiple emulsions are needed so that the aqueous core containing the drug is coated by a polymer shell [65]. Therefore, the majority of published methods use the double emulsion w/o/w solvent evaporation method, with only a few authors reporting on the use of the s/o/w method [11,65]. A schematic illustration of the solvent evaporation method can be found in Figure 3. 

Cao et al. provide an in-depth overview of the influence of various parameters in the w/o/w method on the morphology and physiochemical properties of PLGA particles [73]. In short, the study determined that PLGA concentration and the colostrum emulsification speed are the most influential factors impacting the size of the particles. Lower PLGA concentration levels (10–20 mg/mL) produce particles with a narrow size distribution; however, the shape of the nanoparticles can be rather uneven. A higher PLGA concentration (30–40 mg/mL) allows for the formation of particles with round shapes, although the size distribution of the particles in this case is significantly larger. Up to a certain concentration, an increase in the PLGA concentration increases the drug loading. A higher polymer concentration leads to a higher viscosity and the particles solidify faster. As a consequence, less of the drug can escape from the particles, and the encapsulation efficiency (EE%) and drug loading (DL%) increase [73]. Regarding DL%, the second emulsification step is just as important. Higher emulsification speeds lead to the increased volatilization of solvents like DCM or chloroform, restricting the escape of drugs from particles [73].

Another factor significantly impacting the performance of the formulation method is the pH of the external aqueous phase. Increasing the pH of the external aqueous phase from 5 to 7.4 can increase the EE% up to three times [65]. This phenomenon is based on the deprotonation of the amino groups in the gentamicin molecule, making the molecule less hydrophilic [65].

Poly (vinyl alcohol) (PVA) is the most commonly used surfactant in PLGA-gentamicin nanoformulations. With increasing concentrations of PVA, it is possible to decrease the particle size as well as the polydispersity index (PDI) [66]. A study by Sun et al. suggests that a higher concentration of PVA (9%, 12%) is associated with a more uniform, spherical particle shape [69]. In addition, the hydrophilic nature of PVA could be responsible for the porous surface structures of nanoparticles due to the diffusion processes [69]. The pore size decreases with a higher PLGA concentration [69].

It has been determined that the size of nanoparticles will heavily impact their fate in biological systems. Nanocarriers under 100 nm are prone to endocytosis, while carriers larger than 500 nm will face phagocytosis [74]. The endocytosis of nanoparticles could be more efficient than the pinocytosis of free gentamicin, leading to an increase in the efficiency of the therapy [75]. In practice, most gentamicin-loaded PLGA nanoparticles range between 200 nm and 400 nm (see Table 1). PLGA nanoparticles are internalized using both pinocytosis- and clathrin-mediated endocytosis [76]. After internalization, the PLGA nanoparticles escape the lysosomes and enter the cytoplasm approximately 10 min after incubation [77,78]. It has been suggested that the naturally negative/anionic surface charge of PLGA nanoparticles (due to the carboxylic acid end groups) reverses to cationic with the low pH of endo-lysosomes (pH ≈ 4) [78]. The surface cationization allows for nanoparticles to escape into the cytosol, where the encapsulated drug is released [78]. Moreover, the sustained release of the drug is achievable intracellularly. Since the PLGA nanoparticles are only cationic in the endosomal compartment, and therefore do not cause lysosomal destruction, they could be less toxic in comparison to cationic lipids and cationic polymers [78].

The disadvantages of unmodified PLGA use are the lack of specific targeting properties and response to environmental stimuli, as well as vulnerability to aggregates during freeze-drying [79]. 

Since, without surface modifications, PLGA has no active targeting properties, it has to rely on passive targeting after intravenous administration. Passive targeting will be more effective against conditions that exhibit enhanced permeability and retention effects, such as cancers and inflammatory processes [80]. If no surface modifications to PLGA nanocarriers are applied, opsonization by macrophages usually occurs at high rates [65]. Due to the previously mentioned reasons and the chance of the development of antibacterial resistance, nanoformulations with gentamicin are most often applied for topical or local delivery. 

However, in the case of systemic application, it is possible to apply surface modifications to PLGA to avoid clearance by phagocytes, limit opsonization, and prolong circulation time. The surface of PLGA is most often modified using polyethylene glycol (PEG), different lipids, and target-specific agents, like antibodies and bisphosphonates [81]. To the best of our knowledge, only one publication to date has explored the use of PLGA-PEG for the encapsulation of gentamicin [11].

Three main targets for PLGA–gentamicin nanoparticles can be differentiated—surgical site infections, osteomyelitis, and intracellular infections. 

In the case of osteomyelitis, poor vascularization around bone tissues makes drug delivery to the site of infection very difficult [82]. For both surgical site infections and osteomyelitis, which are commonly caused by *Staphylococcus aureus, Pseudomonas aeruginosa,* and *Escherichia coli*, as well as more resistant strains like *Methicillin-resistant Staphylococcus aureus*, up to 6 weeks of the sustained delivery of gentamicin might be needed to eliminate the infections and their biofilms [66,83,84]. Although non-biodegradable bone cements, like poly (methyl methacrylate) (PMMA), possess several advantages for osteomyelitis treatment, the need for a second surgical intervention to remove them hinders their wider use [85,86]. Biodegradable PLGA-based nanocarrier administration via injection at the site of infection is a great alternative [66].

Another group of infections targeted by gentamicin-loaded PLGA nanoparticles are intracellular infections. Intracellular infections act as a reservoir of bacteria and lead to chronic conditions that can also be lethal. Targeting intracellular infections is much harder due to the poor cellular penetration of gentamicin [64,68]. To overcome this challenge, it is possible to turn to nanoformulations, which, in general, are often taken up by macrophages via phagocytosis. This strategy allows for the passive targeting of intracellular infections and enhances the efficacy of the therapy [68]. To date, gentamicin-loaded PLGA nanoparticles have been effectively explored against two intracellular infections—*Klebsiella pneumoniae* and *Brucella melitensis* [64,68]. Not only can gentamicin-loaded PLGA nanoparticles be taken up by infected macrophages, but they also reduce bacterial viability without inducing an inflammatory or apoptotic response on the macrophages [68]. 

The current evidence of the antibacterial effectiveness of PLGA nanoparticles loaded with gentamicin compared to the use of a free drug is mixed. Empty PLGA nanoparticles do not possess antibacterial properties; therefore, their effect is dependent on the drug content and the drug release mechanisms [65,69]. Some in vitro tests against *P. aeruginosa*, *S. aureus*, and *E. coli* have shown that the MIC and MBC levels of nanoparticles compared to a free drug are equal or one dilution higher [11,65]. Lower effective concentrations of nanoparticles compared to the free drug have been reported against *S. aureus* [11,67]. However, when Jiang et al. tested the use of gentamicin-loaded nanoparticles against *K. pneumoniae* in vitro, the MIC and MBC values for the nanoparticles were significantly higher than those of a free drug [68]. The MIC/MBC values of nanoparticles lowered when tested after a longer period (up to 120 h), while, for the free gentamicin, the values stayed the same. This observation suggests that simple in vitro antibacterial tests might not be suitable for testing nanoparticles with sustained release. Instead, nanoformulations should be tested in more complex physiological environments in vivo for a longer period of time. It was further proven in vivo that gentamicin-loaded nanoparticles were more effective, providing longer protective effects against the infection than the free form of the drug due to the sustained release of the gentamicin from nanoparticles [68]. An increased effectiveness in vivo was also observed with *P. aeruginosa* infection [65]. In addition, in terms of antibiofilm activity, the sustained release of gentamicin proves an advantage over a single dose of free gentamicin [65].

To alleviate the application of prepared nanoparticles and to further modify the release profile of the drugs, it is possible to incorporate the nanoparticles in various other drug delivery forms. For local application, gentamicin-loaded PLGA nanoparticles have been incorporated into transdermal patches and pullulan films [67,87,88].

In sum, the main advantages provided by PLGA nanoparticles are the sustained release of gentamicin for up to 35 days and an enhanced possibility of targeting intracellular infections and infections needing long-term treatment, leading to an increase in their bioavailability. The challenges regarding PLGA–gentamicin nanoparticles are that they require further process optimization to obtain reproducible results, in addition to a quite limited EE%. More studies on the antibacterial efficiency of these nanoparticles using in vivo tests are needed. Gentamicin-loaded PLGA nanoparticles with various surface modifications are an underexplored research field, with future potential for more targeted treatments of infections.

### 4.2. Gentamicin-Loaded Chitosan Nanoparticles

Chitosan is a natural, linear polysaccharide with wide use in the biomedical industry due to its physiochemical properties, biocompatibility, biodegradability, and antibacterial and antifungal activity [89,90]. It is composed of β-1,4 glucose amine and β-1,4-N-acetyl glucose amine units [91]. The antimicrobial properties of chitosan are explained by the electrostatic interaction between the positive charge of chitosan and the negatively charged bacterial cell walls [92,93]. Without the polycationic nature, the antibacterial properties of chitosan are reduced [93]. In addition, other factors, like molecular weight, positive charge density, and pH, can influence the antibacterial properties of chitosan [93,94]. The antimicrobial properties of chitosan make it a desirable polymer for nanoparticle formation and the encapsulation of antibiotics. Gentamicin-loaded chitosan nanoparticles have largely been explored only in the last 5 years. An overview of gentamicin-loaded chitosan nanoparticles and their main parameters can be found in Table 2.

Both physical and chemical crosslinking can be applied to form chitosan nanoparticles [100]. The ionic gelation method, which is a type of physical crosslinking, has been most widely used to form gentamicin-loaded nanoparticles due to the simplicity and mild conditions of the method [96,99]. Sodium tripolyphosphate (TPP), in most cases, is used as a cross-linker. TPP is a non-toxic agent with negatively charged phosphate groups; therefore, it can react with the positively charged amino groups of chitosan, leading to gelling [101,102].

The cellular uptake of chitosan nanoparticles is dependent on their attachment to the negatively charged cell membranes due to electrostatic interactions. The chitosan nanoparticles are then transported via endocytosis, with subsequent release in subcellular compartments like lysosomes [103]. The “proton sponge effect” promotes chitosan nanoparticle escape from endosomes, meaning that the amino groups are more protonated in the acidic pH of endosomes. Subsequently, water and chloride ions enter the endosomes to balance out the osmotic imbalance. The volume increase ruptures the endosomes, allowing the nanoparticles to enter cytoplasm [104]. The same mechanism is also responsible for lysosome rupture [103]. In terms of their possible application, the use of gentamicin-loaded chitosan nanoparticles against intracellular bacteria and biofilms has been examined, as well as explored their potential as wound healing agents [75,95,98,105]. 

Regarding their antibacterial activity, gentamicin-loaded chitosan nanoparticles have shown promising results. Gentamicin-loaded chitosan nanoparticles have shown equal or better antibacterial properties compared to gentamicin in a free drug form [75,97,99,105]. The authors suggest that the higher antibacterial efficiency is observed due to the sustained release of gentamicin [75]. When tested in combination with other agents, like ascorbic acid, the dual-therapy chitosan particles show enhanced antibacterial activity and possible synergy between the components [99].

A disadvantage of using chitosan to encapsulate gentamicin is the positive charge of both molecules, which leads to electrostatic repulsion and, therefore, lower drug loading [95]. In addition, gentamicin release from the nanoparticles is rather short in comparison to that of gentamicin-loaded PLGA nanoparticles, with authors reporting drug release lasting up to 1 week and, in most cases, only around 3 days [75,95,96,97,99,105]. The release profile of chitosan nanoparticles could prohibit their potential use in hard-to-combat infections that require several weeks of treatment. 

In recent years, novel publications can be found on chitosan nanoparticles that combine gentamicin with other active agents, like ascorbic acid, salicylic acid, and proanthocyanidin, to minimize the side effects of gentamicin and enhance the antibacterial activity, with promising results [96,97,99]. As strong antioxidants, these molecules could minimize the toxicity to cells created by reactive oxygen species (ROS) [99].

To further maximize the advantages of both polymeric and lipid drug delivery systems, combined hybrid nanoparticles are currently being researched. In the case of chitosan, Qiu et al. developed novel phosphatidylcholine–chitosan nanoparticles and coated the particles with gentamicin to enhance the drug delivery to biofilms and intracellular pathogens, with promising results [105].

### 4.3. The Release of Gentamicin from Polymeric Nanoparticles

The in vitro release study is crucial in evaluating drug delivery systems’ safety, effectiveness, and quality when utilizing nanoparticles. However, despite its importance, no universally recognized standards or regulations currently govern this type of testing [106,107]. 

Various mechanisms are employed to assess the release of drugs from nanoparticles depending on the drug’s physical and chemical properties and the matrix. These mechanisms include diffusion, erosion, swelling, and osmosis [108,109,110,111,112]. The effectiveness of drug release from biodegradable polymeric microspheres is determined by various factors, including drug loading efficiency, solubility, biodegradability, diffusion, and microsphere size [113].

As highlighted by the literature, gentamicin release studies involving the quantification of gentamicin face several challenges. Firstly, gentamicin is a complex mixture of five structurally distinct components (see Section 2). Secondly, gentamicin lacks chromophore groups, which means that it cannot absorb UV light and can be indirectly determined through chemical derivatization methods with o-phthaldialdehyde, phenyl isocyanate, 9-fluorenylmethyl chloroformate, 1-fluoro-2,4-dinitrobenzene, and others, which are time-consuming and produce unstable derivatives [114,115]. Most of the literature on gentamicin release from PLGA and chitosan mentions derivatization with o-phthaldialdehyde or ninhydrin [11,30,63,65,66,67]. The obtained fluorescent product can be determined through multiple methods. One of the most popular is the more rapid and cost-effective UV/VIS method. Alternatively, the most reliable method is high-performance liquid chromatography (HPLC), which enables UV, fluorescence, electrochemical, or MS detection [114,115].

The drug release profiles of nanoparticle-based formulations can be obtained through three methods: dialysis membrane, sample and separate methods, and the continuous-flow method [106,116,117]. For gentamicin-loaded PLGA and chitosan nanoparticles, the most commonly used methods are the first two.

One of the major challenges in comparing research findings in the field of gentamicin release is the absence of standardization in experimental methods. This leads to variations in the type of membranes used, stirring speed (50–200 rpm), medium (NaCl or PBS with pH 6.4–7.4), temperature (37 °C or 32 °C), and volume (500–900 mL) [11,30,63,65,66,67,69]. Even when the same techniques, such as UV/VIS, are employed, different wavelengths are often used to detect fluorescence or absorbance [11,30,69]. Fluorescence has been measured at 360/460 nm [63,65,68]. UV absorbance has been detected at 332 nm [66], 334 nm [75], 335 nm [67], and 400 nm [11]. Due to the variability in drug release profiles (Higuchi, first or zero order), it can be challenging to compare the published results and draw meaningful conclusions. This can lead to different interpretations of the drug release times and concentrations [69,113,118].

## 5. Hydrophobization of the Gentamicin Molecule

**Figure 4:**
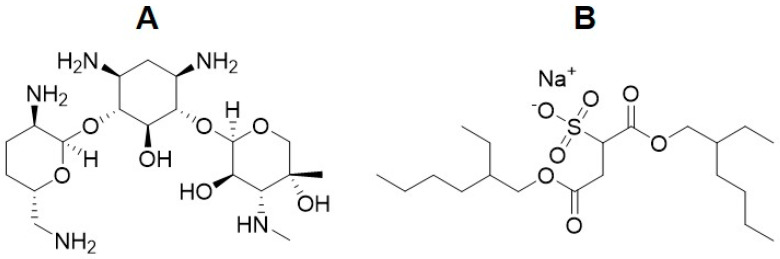
Structures of (**A**): gentamicin; (**B**): docusate sodium salt (AOT).

To increase the efficacy of antimicrobial drug activity against various infectious diseases, it is necessary to increase antimicrobial activity in the intracellular environment or prolong the presence of the antibiotic to produce a therapeutic effect. Gentamicin sulfate is a polar antimicrobial drug and, due to its hydrophilic nature, penetration into infected cells is limited. Moreover, as gentamicin shows a concentration-dependent bactericidal activity and post-antibiotic effect, it requires regular and high doses, which lead to increases in side effects [31]. Nanotechnology has developed a promising approach for the treatment of intracellular infections by providing the intracellular targeting and sustained release of encapsulated drugs inside the infected cells [119]. Initially, hydrophilic gentamicin sulfate was encapsulated in PLGA particles, and its encapsulation efficiency was achieved at 19.2% at 9.2 µg/mg of the polymer. However, drug release tests revealed that most of the gentamicin was released within the first hour. This can be explained by the possible absorption of gentamicin on the surface of PLGA particles [63]. Therefore, a highly efficient drug encapsulation strategy is required. The hydrophobic ion pairing of gentamicin with anionic surfactants without losing antimicrobial activity serves well for this purpose [120]. This method is well known to convert polar drugs into non-polar complexes and improve their encapsulation efficiency. For this purpose, active substances such as docusate sodium salt (AOT), sodium dodecyl sulfate, sodium oleate, and sodium deoxycholate sulfate are used [121]. Docusate sodium salt is one of the most promising anionic surfactants for gentamicin. The chemical structure of gentamicin and AOT are presented in Figure 4. 

The ionic complex of the Gent–AOT ratio is 1:5, and the stoichiometric complexation of the five ionizable amino groups of gentamicin is neutralized with the AOT sulfate group. The lipophilic alkyl chains of AOT make this complex soluble in organic solvents [122]. The obtained complexes form particles with a size of 1000 nm [123], followed by further encapsulation of the hydrophobic Gent–AOT complex in a biodegradable water-insoluble polymer (PLGA), making it possible to obtain nanoparticles with a size of 200–300 nm [122,123,124]. This strategy allows for a gentamicin encapsulation efficacy close to quantitative yields and up to 60 µg/mg of the PLGA polymer compared to instances where sulfate is used as an ion pairing agent [125]. In addition, encapsulated gentamicin is released from PLGA particles much more slowly in drug release tests due to the hydrophobic gentamicin AOT complex. It was observed that only 10% of gentamicin was released during the first hour, but an almost linear sustained release of the drug over 10 weeks was observed later [122]. Other biodegradable polymers are also used for the encapsulation of hydrophobic Gent–AOT, for example, poly (ε-caprolactone) and poly (D,L-lactic acid) [126], polyvinyl alcohol [122], poly (aspartic acid) [127] or poly (methyl vinyl ether-co-maleic anhydride) [128]. The variation in surfactants is another tool to construct a drug delivery system, as the interactions of the gentamicin complex with the AOT that was entrapped into PLGA are known to provide a more efficient drug delivery than occurs in the absence of AOT [31].

The antibacterial properties of nanoparticles that contain Gent–AOT are superior. However, the toxicity of Gent–AOT should also be considered, and more research is needed to evaluate gentamicin nanoformulations [124,125].

## 6. Gentamicin Combinations with Other Antibiotics

Early studies started investigating the synergic effects of gentamicin, together with other antibiotics, like β-lactams, in the 1970s, reporting better outcomes for the combination approach [129,130,131]. Currently, gentamicin is co-prescribed with a range of penicillins (amoxicillin, ampicillin, and benzylpenicillin) for the treatment of serious infections like sepsis, bacterial pneumonia, and peritoneal abscess [8]. The World Health Organization recommends gentamicin + azithromycin dual treatment for gonococcal infections in case treatment with cephalosporins fails, although this is a conditional recommendation with very low-quality evidence [132]. In the last decade, several studies have searched for new potential synergic combinations. Synergy has been observed when combining gentamicin with azithromycin [133,134], mitomycin C [135], fosfomycin [136,137], ciprofloxacin [136], daptomycin [138], and cefepime [139].

Recent tests on gentamicin in combination with other antibiotics have largely focused on its antibacterial activity against *P. aeruginosa* [134,135,136,139] and *Enterococci* species [137,138]. In addition, the combinational approach has also been tested against uncomplicated gonorrhea [133] and *E. coli* strains [136]. The synergic effects of combinations against biofilms of both *E. coli* (fosfomycin/gentamicin [136]) and *P. aeruginosa* (ciprofloxacin/gentamicin [136] and cefepime/gentamicin [139]) may be particularly important. A fosfomycin/gentamicin combination even retained its synergic effects against a gentamicin-resistant strain of *E. coli* [136]. 

The mechanisms behind the synergic effects of the two antibacterial agents are still largely unknown and underexplored. It has been suggested that the synergy of the azithromycin/gentamicin combination might be due to the suppression of trans-translation by gentamicin, enhancing the efficacy of azithromycin [134]. In addition, a bacterolytic effect of gentamicin has been hypothesized to explain the efficacy of the fosfomycin/gentamicin combination against a gentamicin-resistant strain [136]. Due to the high clinical significance, understanding the mechanism behind the synergic effects responsible for action against biofilms is critical.

Reports have been presented on the increased development of resistance after using the gentamicin combination compared to monotherapy [140]. Furthermore, the increased toxicity, especially the nephrotoxicity, of this approach is particularly concerning [29,140,141,142]. For example, a study of patients with gonorrhea revealed a strong adverse gastrointestinal effect rate in patients, which would likely limit the application of the azithromycin/gentamicin combination in clinical practice [133].

Further studies, including randomized controlled trials, should be executed to evaluate the risks and benefits of gentamicin + other antibiotic approaches. A search for alternative antibacterial agents, which have safer profiles and a lower likelihood of the development of resistance, like substances derived or isolated from natural products, is needed.

## 7. Gentamicin Combinations with Polyphenols

The used combination should lower the effective dose of antibiotic, as well as minimize the potential side-effects of the treatment [2,143]. These criteria could potentially be met by natural products like individual phytochemicals or herbal extracts. 

Polyphenols are one of the largest groups of natural bioactive substances and are present in a very wide variety of plants [144]. Polyphenols possess not only antimicrobial but also antioxidant and anti-inflammatory properties [144,145]. Even though the effective concentrations of polyphenols often are much higher than those of antibiotics, their overall properties and potential for synergism make them attractive candidates for the combination approach [146]. 

A wide range of polyphenols, mainly flavonoids, have been tested together with gentamicin in the last 20 years. For the most part, more flavonoid aglycones than glycosides have been tested. Aglycones are known to have more potent biological activity than their respective glycosides [147]. An overview of studies with positive conclusions regarding synergy is found in Table 3. Polyphenols that, to date, have not shown any synergistic effects together with gentamicin have not been included in Table 3. 

Three main mechanisms of polyphenol and gentamicin synergy can be distinguished. First, polyphenols inhibit the bacterial wall biosynthesis, allowing gentamicin to reenter the cells and leading to increased sensitivity [146,152,153]. Second, polyphenols can inhibit different bacterial efflux pumps; for example, AbeM and AdeABC [146,153]. Since these efflux pumps can be responsible for the development of resistance, their inhibition could increase bacterial sensitivity against antibiotics [146]. Third, polyphenols can inhibit quorum sensing, consequently regulating the bacterial population density [2,155,158]. Quorum sensing plays a significant role in biofilm production, hindering optimal drug delivery to pathogens. The inhibition of quorum sensing disrupts the biofilm structure, allowing gentamicin to reach and kill the pathogens [2,153,155]. 

Overall, most of the research focused on members of the ESKAPE group of pathogens, providing potential future alternatives to current approaches. To date, the lowest amount of evidence of synergy was found for *E. coli*, with only two papers finding synergy or additive effects using reference strains [3,143], and the rest reporting no or minimal effect [146,148,154,159].

In terms of the tested bacterial strains, it is advisable to use not only commercially available reference strains but also clinical isolates, since different strains may exhibit different synergic effects due to their diverse genetic and resistance mechanisms [2]. The synergic activity of polyphenols with gentamicin against reference strains does not always translate to clinical isolates [143]. 

In addition to the synergic effects, polyphenols have also been investigated to minimize the toxic effects of oxidative stress subsequent to the use of gentamicin [143,159].

## 8. Gentamicin Combinations with Natural Products

Another approach to enhance the antibacterial properties of gentamicin is to test multicomponent natural products like herbal extracts. Herbal extracts contain up to hundreds of bioactive substances and, therefore, could simultaneously act on multiple targets and enhance each other’s antibacterial activity [160]. In addition, the use of multi-component products could lower the chances of antibacterial resistance development [161]. An overview of studies with positive conclusions regarding the synergy between natural products and gentamicin is found in Table 4. Natural products that, to date, have not shown any synergistic effects together with gentamicin are not included in Table 4.

Most positive outcomes regarding the synergy between natural products and gentamicin were obtained using bacterial reference strains, raising the question of whether the same observation could be seen using clinical isolates. If using medicinal plant extracts, the preparation method of the extract is crucial to the chemical composition of the extract. Furthermore, the wide variability in the chemical profiles and concentrations of bioactive substances in plants has to be kept in mind. Therefore, studies on the potential synergy between herbal extracts and antibiotics should also provide results on the chemical composition of the extract. 

A concrete conclusion regarding the mechanisms responsible for the synergic activity of natural products together with antibiotics cannot be obtained [161,162,163,164]. Some authors have suggested that synergic effects could be observed due to changes in the efflux system, damage to cell membranes, and the inhibition of protein synthesis. Further research is needed to clearly understand the mechanisms [161,162,164]. A study using propolis revealed that the concentration of natural products is also crucial to the synergic activity [161]. Higher concentrations of propolis and antibiotics lead to a surprising loss of synergy [161]. This finding accentuates the important role of the ratio of components to produce synergic activity [161].

**Table 4:** Synergy between gentamicin and natural products.

Natural Product	Bacteria	Bacterial Strain Type	Antibacterial Effect on Gentamicin	FICI	Synergy/Partial Synergy	Ref.
*Aniba rosaeodora* essential oil	*B. cereus*	Reference	MIC reduction from 0.50 μg/mL to 0.12 μg/mL	0.30	Synergy	[40]
	*B. subtilis*	Reference	MIC reduction from 0.25 μg/mL to 0.06 μg/mL	0.34	Synergy	[40]
	*S. aureus*	Reference	MIC reduction from 0.50 μg/mL to 0.12 μg/mL and from 0.06 μg/mL to 0.01 μg/mL	0.30	Synergy	[40]
	*E. coli*	Reference	MIC reduction from 0.50 μg/mL to 0.12 μg/mL	0.35	Synergy	[40]
	*A. baumannii*	Reference	MIC reduction from 4.00 μg/mL to 0.24 μg/mL	0.11	Synergy	[40]
	*S. marcescens*	Reference	MIC reduction from 0.50 μg/mL to 0.12 μg/mL	0.30	Synergy	[40]
	*Y. enterocolitica*	Reference	MIC reduction from 0.25 μg/mL to 0.01 μg/mL	0.11	Synergy	[40]
*Clinopodium vulgare* L. extracts	*B. subtilis*	Clinical isolates	Reduction in MIC	0.395–0.44	Synergy	[164]
*Daphne genkwa* extract	*S. aureus* (methicillin-resistant)	Reference	Reduction in MIC	0.750	Partial synergy	[165]
*Magnolia officinalis* extract	*S. aureus* (methicillin-resistant)	Reference	Reduction in MIC	0.750	Partial synergy	[165]
*Kaempferia parviflora* extracts	*K. pneumoniae*	Clinical isolates	Reduction in MIC	0.141–0.625	Synergy/Partial synergy	[162]
	*P. aeruginosa*	Clinical isolates	Reduction in MIC	0.133–0.625	Synergy/Partial synergy	[162]
	*A. baumannii*	Clinical isolates	Reduction in MIC	0.133–0.563	Synergy/Partial synergy	[162]
*Mentha piperita* L. essential oil	*B. cereus*	Reference	MIC reduction from 2.00 μg/mL to 0.06 μg/mL	0.08	Synergy	[163]
	*B. subtilis*	Reference	MIC reduction from 0.50 μg/mL to 0.01 μg/mL	0.07	Synergy	[163]
	*S. aureus*	Reference	MIC reduction from 2.0 μg/mL to 0.06 μg/mL; from 0.5 μg/mL to 0.06 μg/mL; from 8.0 μg/mL to 2.0 μg/mL	0.103–0.3	Synergy	[163]
	*E. faecalis*	Reference	MIC reduction from 8.0 μg/mL to 1.0 μg/mL	0.32	Synergy	[163]
	*E. coli*	Reference	MIC reduction from 1.0 μg/mL to 0.03 μg/mL	0.43	Synergy	[163]
	*K. pneumoniae*	Reference	MIC reduction from 32.0 μg/mL to 1.0 μg/mL	0.43	Synergy	[163]
	*A. baumannii*	Reference	MIC reduction from 8.00 μg/mL to 0.5 μg/mL	0.46	Synergy	[163]
	*P. aeruginosa*	Reference	MIC reduction from 2.00 μg/mL to 0.06 μg/mL	0.08	Synergy	[163]
*Pelargonium graveolens* essential oil	*B. cereus*	Reference	MIC reduction from 0.50 μg/mL to 0.125 μg/mL	0.30	Synergy	[40]
	*B. subtilis*	Reference	MIC reduction from 0.25 μg/mL to 0.06 μg/mL	0.34	Synergy	[40]
	*S. aureus*	Reference	MIC reduction from 0.50 μg/mL to 0.01 μg/mL and from 0.12 to 0.04 μg/mL	0.28–0.35	Synergy	[40]
	*E. coli*	Reference	MIC reduction from 0.50 μg/mL to 0.12 μg/mL	0.30	Synergy	[40]
	*A. baumannii*	Reference	MIC reduction from 4.00 μg/mL to 0.24 μg/mL	0.11	Synergy	[40]
	*S. marcescens*	Reference	MIC reduction from 0.50 μg/mL to 0.12 μg/mL	0.45	Synergy	[40]
	*Y. enterocolitica*	Reference	MIC reduction from 0.25 μg/mL to 0.03 μg/mL	0.22	Synergy	[40]
Propolis	*B. subtilis*	Reference	MIC reduction from 1.25 μg/mL to 0.05 μg/mL	NA	Synergy	[161]
	*B. cereus*	Reference	MIC reduction from 1.25 μg/mL to 0.05 μg/mL	NA	Synergy	[161]
	*B. megaterium*	Reference	MIC reduction from 0.25 μg/mL to 0.01 μg/mL	NA	Synergy	[161]
	*S. aureus* (methicillin sensitive)	Reference	MIC reduction from 1.5 μg/mL to 0.5 μg/mL	NA	Synergy	[161]
	*S. aureus* (methicillin-resistant)	Reference	MIC reduction from >1.5 μg/mL to 0.5 μg/mL	NA	Synergy	[161]
	*E. coli*	Reference	MIC reduction from >1.5 μg/mL to 0.5 μg/mL	NA	Synergy	[161]

NA—not available; FICI—fractional inhibitory concentration index; MIC—minimum inhibitory concentration.

## 9. Conclusions and Future Perspectives

Even after 60 years on the market, gentamicin plays an important role in combating serious infections, especially those originating from the nosocomial ESKAPE pathogens. Several strategies, including encapsulation in nanoformulations, hydrophobization of the molecule, and combinations with various agents, have been investigated to enhance both the activity and drug delivery of gentamicin. Current evidence indicates that polymeric nanoformulations, especially PLGA nanoparticles, could provide a sustained release of gentamicin and offer higher bioavailability. Hydrophobization of the gentamicin molecule can significantly increase the encapsulation efficiency in nanoparticles and enhance the antibacterial properties of the system. Nevertheless, the currently used in vitro drug release methods lack standardization, hindering adequate comparison between studies. Further research on the toxicology of these nanoformulations is needed. Research on the synergic effects between gentamicin and polyphenols is an exciting research direction. Testing on a wider range of clinical isolates, not only reference strains of bacteria, will allow us to see if the results of synergy can be translated to clinical practice. The possible encapsulation of gentamicin–polyphenol combinations in drug delivery systems should be further investigated. As gentamicin is applied to treat hard-to-reach infections like osteomyelitis, target-specific surface modifications of drug delivery systems hold great potential for future research. 

## Figures and Tables

**Table 1:** Gentamicin-loaded PLGA nanoparticles.

PLGA Type	Surfactant	Formulation Method	Size (nm)	PDI	Zeta Potential (mV)	Particle Characterization Methods	EE (%)	Drug Loading	Drug Release (In Vitro)	Bacteria (In Vitro Tests)	Potential Application	Ref.
50:50 (13.7 kDa)	PVA (15 kDa)	w/o/w solvent evaporation	320	NA	−15.5 ± 0.2	DLS ELS SEM XRD DSC	13.12–58.76	3.24–7.35 ^#^	28 days	NT	Intracellular pathogens	[63]
50:50 (13.7 kDa)	PVA (15 kDa)	w/o/w solvent evaporation	310 ± 2.00	NA	NT	DLS	NT	6.2 *	NT	NT	Intracellular pathogens	[64]
50:50 (12 kDa)	PVA	w/o/w s/o/w solvent evaporation	241.3–358.5	0.10–0.23	−0.4–2.3	DLS ELS	NT	6.4–22.4 *	Over 16 days	*P. aeruginosa*	Planktonic- and biofilm-based infections	[65]
85:15 (80 kDa)	PVA (31 kDa)	w/o/w solvent evaporation	219–391	0.21–0.38	−7.2–−1.1	DLS ELS AFM	2.7–52.4	0.06–10.28%	35 days	*S. aureus* *S. epidermidis*	Osteomyelitis	[66]
50:50 (7–17 kDa)	PVA (85–124 kDa)	w/o/w solvent evaporation	280 ± 12.04	0.15 ± 0.01	−4.9 ± 0.84	DLS ELS SEM TEM	NT	60%	216 h	*P. aeruginosa* *S. aureus*	Surgical site infections	[67]
50:50 (7–17 kDa)	PVA	w/o/w solvent evaporation	227	0.162	−1.67	DLS ELS SEM	NT	135 *	120 h	*K. pneumoniae*	Intracellular pathogens	[68]
PLGA-PEG (70 kDa)	PVA (85–124 kDa)	s/o/w solvent evaporation	140.0–919.3	0.104–1.230	−5.54–0.36	DLS ELS TEM	43.97–64.61	2.9–7.9%	10 h	*P. mirabilis* *E. coli* *P. aeruginosa* *S. aureus*	Intracellular pathogens	[11]
75:25 (4–15 kDa)	PVA (89–98 kDa)	w/o/w solvent evaporation	32–2400	NA	NT	DLS SEM	NT	NT	10 h	*E. coli*	Wound treatment	[69]

^#^—µg gentamicin/mg particles; *—µg gentamicin/mg polymer; NA—not available; NT—not tested; PVA—poly (vinyl alcohol); PDI—polydispersity index; DLS—dynamic light scattering; ELS—electrophoretic light scattering; AFM—atomic force microscopy; TEM—transmission electron microscopy; SEM—scanning electron microscopy; DSC—differential scanning calorimetry; XRD—X-ray diffraction analysis.

**Table 2:** Gentamicin-loaded chitosan nanoparticles.

Chitosan Type	Crosslinker	Drugs	Formulation Method	Size (nm)	PDI	Zeta Potential (mV)	Particle Characterization Methods	EE (%)	Drug Loading (%)	Bacteria (In Vitro Tests)	Potential Application	Ref.
150 kDa, deacetylation degree 85.6%	TPP	Gentamicin	Ionic gelation	779.37 ± 51.79	NT	1.9 ± 0.5	DLS ELS	78.06 ± 2.13	63.10 ± 1.54	NT	Intracellular pathogens	[95]
80 kDa, deacetylation degree 95%	TPP	Gentamicin + salicylic acid	Ionic gelation	148–345	0.234–0.428	32.45–42.43	DLS ELS TEM SEM FTIR XRD	61.70–87.20	13.56–26.64	NT	Reduction in toxicity	[96]
140 kDa, deacetylation degree 85%	TPP	Gentamicin	Ionic gelation	100	NT	28	DLS ELS SEM	72	22	*B. abortus* *B. melitensis*	Intracellular pathogens	[75]
304 kDa, deacetylation degree >84%	-	Gentamicin + proanthocyanidin	Hydrogen bonding	242.9–277.4	0.344–0.391	34.5–38.5	DLS ELS SEM FTIR TGA	94	NT	*E. coli* *S. aureus* *P. aeruginosa*	Enhanced antimicrobial activity	[97]
Low molecular weight, deacetylation ≥ 75%	TPP	Gentamicin	Ionic gelation	151–212	0.21–0.29	37.2–51.1	DLS ELS TEM SEM DSC	36.6–42.7	NT	NT	Wound healing	[98]
NA	TPP	Gentamicin + ascorbic acid	Ionic gelation	278	NT	30.01	DLS ELS TEM FTIR	89	22	*S. aureus* *P. aeruginosa*	Reduction in toxicity	[99]

NA—not available; NT—not tested; TPP—sodium tripolyphosphate; DLS—dynamic light scattering; ELS—electrophoretic light scattering; TEM—transmission electron microscopy; SEM—scanning electron microscopy; TGA—thermogravimetric analysis; DSC—differential scanning calorimetry; FTIR—Fourier-transform infrared spectroscopy; XRD—X-ray diffraction analysis.

**Table 3:** Synergy between gentamicin and individual polyphenols.

Polyphenol	Bacteria	Bacterial Strain Type	Antibacterial Effect on Gentamicin	FICI	Synergy/Partial Synergy	Ref.
Caffeic acid	*P. aeruginosa*	Clinical isolates	MIC reduced from 625 μg/mL to 24.61 μg/mL	NA	Synergy	[148]
Epigallocatechin gallate	*A. baumannii*	Reference	MIC reduced from 27 μg/mL to 4 μg/mL	0.65	Partial synergy	[146]
	*S. aureus*	Clinical isolates	MIC reduced from 32 μg/mL to 6.4 μg/mL	0.325	Synergy	[149]
	*E. coli*	Clinical isolates	MIC reduced from 32 μg/mL to 6.4 μg/mL	0.325	Synergy	[149]
Daidzein	*A. baumannii*	Reference	MIC reduced from 27 μg/mL to 8 μg/mL	0.42	Synergy	[146]
Galangin	*S. aureus* (methicillin-resistant)	Clinical isolates/reference	Reduced MIC	0.18–0.25	Synergy	[150]
Gallic acid	*S. aureus*	Clinical isolates	MIC reduced from 49.21 μg/mL to 2.44 μg/mL	NA	Synergy	[148]
Genistein	*A. baumannii*	Reference	MIC reduced from 27 μg/mL to 4 μg/mL	0.4	Synergy	[146]
5-Hydroxy-3,7,4′-trimethoxyflavone	*S. aureus*	Clinical isolates	Reduced MIC	NA	Synergy	[151]
	*E. coli*	Clinical isolates	Reduced MIC	NA	Synergy	[151]
Kaempferol 7-O-β-D-(6″-O-cumaroyl)-glucopyranoside	*S. aureus*	Reference	MIC reduced from 16 μg/mL to 4 μg/mL	NA	Synergy	[3]
	*E. coli*	Reference	MIC reduced from 16 μg/mL to 8 μg/mL	NA	Synergy	[3]
Luteolin	*S. aureus*	Reference	MIC reduced 4-fold	0.258	Synergy	[143]
	*E. coli*	Reference	Reduced MIC	0.504	Additive	[143]
Nordihydroguaiaretic acid	*S. aureus* (methicillin-sensitive)	Clinical isolates	Several-fold MIC reduction	<0.5	Synergy	[152]
	*S. aureus* (methicillin-resistant)	Clinical isolates	Several-fold MIC reduction	<0.5	Synergy	[152]
Quercetin	*P. aeruginosa*	Clinical isolates	MIC reduced from 128 μg/mL to 32 μg/mL	0.28–0.53	Synergy/Partial synergy	[153]
	*P. aeruginosa*	Clinical isolates/reference	Reduced MIC	0.375–0.75	Synergy/Partial synergy	[2]
	*P. mirabilis*	Clinical isolates	Restored antibacterial activity	NA	Synergy	[154]
Plumbagin	*P. aeruginosa*	Reference	Reduced MIC	0.152–0.485	Synergy	[155]
Pyrogallol	*S. aureus*	Clinical isolates	MIC reduced from 49.21 μg/mL to 2.44 μg/mL	NA	Synergy	[148]
Rutin	*P. aeruginosa*	Reference	MIC reduced from 10 μg/mL to 2.5 μg/mL	0.5	Synergy	[156]
Sophoraflavanone B	*S. aureus* (methicillin-resistant)	Clinical isolates/reference	MIC reduced 8- to 32-fold	0.25–0.375	Synergy	[157]
Vitexin	*P. aeruginosa*	Reference	Reduced MIC	0.078	Synergy	[158]

NA—not available; FICI—fractional inhibitory concentration index; MIC—minimum inhibitory concentration.

## Data Availability

Not applicable.
